# A Modified Fenton’s System Fe^2+^–EGTA–H_2_O_2_ Reveals That Redox Activities of Simple Polyphenols Are Suppressed in Binary Mixtures

**DOI:** 10.3390/molecules30112269

**Published:** 2025-05-22

**Authors:** Michał Nowak, Krzysztof Sasak, Anna Wlodarczyk, Izabela Grabska-Kobylecka, Agata Sarniak, Dariusz Nowak

**Affiliations:** 1 Radiation Protection, University Hospital No. 2, Medical University of Lodz, Zeromskiego 113, 90-549 Lodz, Poland; m.nowak@skwam.lodz.pl; 2 Department of Medical Imaging Techniques, Medical University of Lodz, Lindleya 6, 90-131 Lodz, Poland; krzysztof.sasak@umed.lodz.pl; 3 Department of Sleep Medicine and Metabolic Disorders, Medical University of Lodz, Mazowiecka 6/8, 92-215 Lodz, Poland; anna.wlodarczyk@umed.lodz.pl; 4 Department of Clinical Physiology, Medical University of Lodz, Mazowiecka 6/8, 92-215 Lodz, Poland; izabela.grabska-kobylecka@umed.lodz.pl (I.G.-K.); agata.sarniak@umed.lodz.pl (A.S.)

**Keywords:** chemiluminescence, Fenton system, plant phenolic acids, polyphenol additive effect, antioxidant, pro-oxidant activity

## Abstract

Various polyphenols are absorbed into the bloodstream following the consumption of polyphenol-rich foods. These compounds may exhibit divergent redox properties, particularly in relation to their antioxidant or pro-oxidant activities. We evaluated the effects of six binary equimolar combinations of polyphenols with pre-established redox profiles on hydroxyl radical-dependent ultra-weak photon emission (UPE) in an Fe^2+^–EGTA–H_2_O_2_ Fenton system: gallic acid and vanillic acid, gallic acid and 3,4-dihydroxyphenylacetic acid, gallic acid and homovanillic acid, ellagic acid and 3,4-dihydroxyphenyl acetic acid, ellagic acid and homovanillic acid, and vanillic acid and homovanillic acid. The first pair of phenolics gave the expected summed redox response. The second one gave a higher redox response than expected from the sum (512 ± 461% vs. 212 ± 222% of enhancement of UPE, *p* < 0.05). The remaining four pairs revealed a lower redox response than expected from the sum (*p* < 0.05). The biggest difference was found for elagic acid and homovanillic acid (357 ± 50% vs. 1689 ± 293% enhancement). These findings suggest that the predictive value of individual redox profiles of phenolics is limited for the calculation of the experimental effect of their binary mixtures on the UPE of the Fe^2+^–EGTA–H_2_O_2_ system. We hypothesize that polyphenol polymerization may be responsible for this phenomenon.

## 1. Introduction

### 1.1. Plausible Antagonism of Plant Polyphenols in Relation to Health-Promoting Activities

Fruits and vegetables represent a major dietary source of phytochemicals, notably vitamins and polyphenols, which exhibit a wide range of bioactivities. To date, over 8000 distinct plant-derived polyphenols have been identified and structurally characterized [1]. Following the consumption of polyphenol-containing meals, various phenolic compounds and their microbial or enzymatic metabolites enter systemic circulation [2,3], potentially modulating intracellular pathways in multiple tissues. A single fruit or vegetable may contain dozens of structurally diverse polyphenolic species [2,3,4]. These compounds exert a broad spectrum of physiological effects, including anti-diabetic, antioxidant, anti-cancer, anti-aging, and anti-atherogenic actions. However, the biological activities of polyphenols are highly compound-specific and, in certain contexts, they may elicit antagonistic or even opposing intracellular effects including mutagenic, carcinogenic, or genotoxic properties [5]. Antagonistic interactions have been described between specific polyphenols, such as chlorogenic acid from coffee and various cardamom-derived phenolics (e.g., protocatechuic acid, vanillic acid, p-coumaric acid, ferulic acid), particularly with respect to metal-chelating capacity and lipoxygenase inhibition [6]. Depending on the assay employed, the same polyphenol may yield markedly different results [7], and several studies suggest that some polyphenols may act as pro-oxidants under physiological conditions [8,9,10].

### 1.2. Examples of Mutual Polyphenols Interactions Evaluated In Vitro with Redox Activity Assays

In support of this, recent studies employing ORAC (Oxygen Radical Absorbance Capacity) and FRAP (Ferric Reducing Antioxidant Power) assays have demonstrated that mixtures of phenolic acids—prepared as binary, ternary, quaternary, and quinary equimolar combinations—exhibit synergistic, antagonistic, or non-additive antioxidant activities when compared to the sum of their individual predefined values [11]. Comparable findings were reported using the ABTS assay, which measures the capacity of antioxidants to scavenge the ABTS•^+^ radical cation. Mixtures of gallic acid, ferulic acid, and caffeic acid demonstrated marked antagonism, attributable not to direct chemical interaction between the phenolics, but rather to differences in reaction kinetics with the ABTS•^+^ radical [12].

### 1.3. Modified Fenton’s System Fe^2+^–EGTA–H_2_O_2_ as a Tool for Determination of Polyphenol Interactions and Aims of the Study

In a recent study, we evaluated the antioxidant and pro-oxidant behavior of 17 plant-derived polyphenols using a modified Fenton system consisting of ferrous ions (Fe^2+^), EGTA (ethylene glycol-bis(β-aminoethyl ether)-N,N,N′,N′-tetraacetic acid), and hydrogen peroxide (H_2_O_2_) [13]. In this system, hydroxyl radicals (•OH) generated by the Fenton reaction cleave ether bonds in the EGTA backbone, leading to the formation of triplet-excited carbonyl intermediates [14]. The radiative decay of these intermediates produces ultra-weak photon emission (UPE), the attenuation of which serves as a quantitative proxy for anti-•OH activity [14]. Eight compounds demonstrated significant •OH-scavenging activity, five were pro-oxidant, and four (ferulic acid, chlorogenic acid, resorcinol, and cyanidin-3-O-glucoside) displayed concentration-dependent redox switching [13]. This raises several fundamental questions: Do binary mixtures of polyphenols with known pro- or antioxidant activity yield additive, synergistic, or antagonistic effects in •OH-generating systems? Can the pro-oxidant effect of one compound negate the antioxidant potential of another? Is mutual augmentation possible among structurally or functionally related phenolics? To address these questions, we investigated the effects of six binary polyphenol mixtures comprising gallic acid, ellagic acid, vanillic acid, homovanillic acid, and 3,4-dihydroxyphenylacetic acid on the chemiluminescent output of the Fe^2+^–EGTA–H_2_O_2_ system, used here as a proxy for •OH radical activity in vitro.

## 2. Results

**Figure 1:**
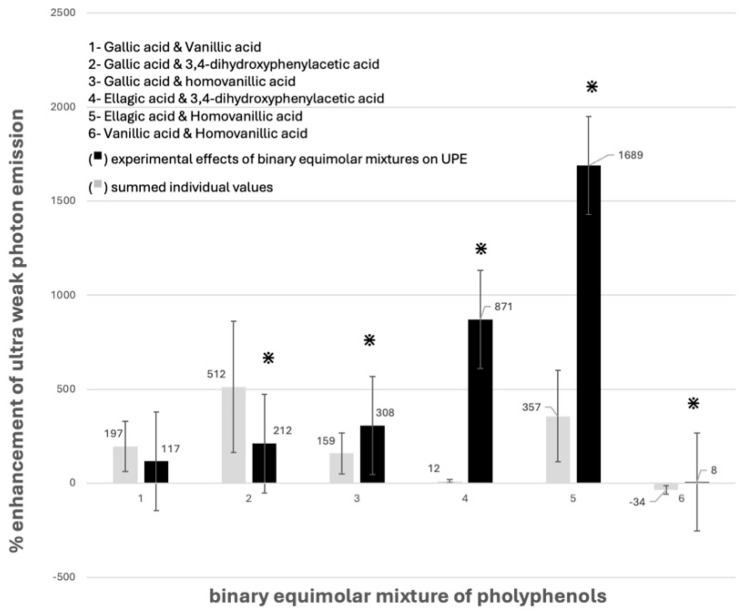
Comparison of experimental and summed individual values effects of binary equimolar mixtures of polyphenols on light emission (UPE) generated by the Fe^2+^–EGTA–H_2_O_2_ system. Results expressed as mean and standard deviation of % enhancement (+) or inhibition (−) of light emission were obtained from at least five separate experiments. *—versus corresponding experimental value—*p* < 0.05.

Figure 1 and Table S1 summarize the effects of five individual polyphenols—gallic acid, ellagic acid, vanillic acid, homovanillic acid, and 3,4-dihydroxyphenylacetic acid—as well as the experimental and summed individual values outcomes of six binary equimolar mixtures composed of these compounds (gallic acid and vanillic acid, gallic acid and 3,4-dihydroxyphenylacetic acid, gallic acid and homovanillic acid, ellagic acid and 3,4-dihydroxyphenyl acetic acid, ellagic acid and homovanillic acid, and vanillic acid and homovanillic acid) on •OH-dependent ultra-weak photon emission (UPE) in the modified Fe^2+^–EGTA–H_2_O_2_ Fenton system.

To ensure maximal relevance of the redox profiles of the studied compounds, each experiment was performed using two individual polyphenols and their equimolar mixture, alongside appropriate negative and system-incomplete controls. Among the five polyphenols, three (gallic acid, ellagic acid, and homovanillic acid) exhibited clear pro-oxidant activity, as evidenced by enhanced UPE compared to the control without added polyphenol. In contrast, 3,4-dihydroxyphenylacetic acid demonstrated antioxidant properties by significantly inhibiting UPE in comparison to that generated by Fe^2+^–EGTA–H_2_O_2_, whereas vanillic acid showed a neutral effect under the tested conditions (Table S1). These findings differ from those reported in our previous work, where vanillic and homovanillic acids displayed marked antioxidant properties revealed by inhibition of light emission from the complete system [13]. A plausible explanation for this discrepancy lies in the difference in the pH of the reaction milieu; the previous study employed a pH of 7.4, corresponding to human blood [13], whereas the current investigation utilized a pH of 6.6, mimicking intracellular conditions. It has been shown that variations in pH can significantly influence the redox behavior of polyphenols [15], and in our earlier work we demonstrated that both UPE intensity and the UPE-to-noise ratio peaked at pH 6.6 when stabilized with 10 mmol/L phosphate buffer [16]. Control samples (incomplete systems, with and without polyphenols) displayed low and stable baseline emissions, consistent with our earlier observations [13,14]. Among the six equimolar binary combinations, only one pair, vanillic acid and gallic acid, exhibited no statistically significant difference (*p* > 0.05) between the summed individual values and experimental impact on UPE. In contrast, the remaining five mixtures revealed significant discrepancies (*p* < 0.05), with the experimental value less than the summed value for individual phenolics in four cases, suggesting that combining polyphenols diminishes their antioxidant activity. One pair had higher redox activity than the summed value for its individual phenolics, suggesting enhanced antioxidant activity for this combination. Notably, the vanillic acid and homovanillic acid combination showed a reversal in effect; while the summed individual prediction indicated an enhancement, the observed experimental result revealed a net inhibitory action on •OH-dependent UPE of Fe^2+^–EGTA–H_2_O_2_ system. These outcomes suggest that chemical interactions may occur between polyphenolic compounds themselves, or between polyphenols and constituents or reaction products of the Fenton system (e.g., Fe^2+^, Fe^3+^, or reactive oxygen species), thereby modulating or suppressing their inherent pro- or antioxidant activities.

## 3. Discussion

Although the modified Fenton system employed in our study, comprising only Fe^2+^, EGTA, and H_2_O_2_, was relatively simple, no additive effect on •OH-induced UPE was observed in five out of six tested binary phenolic combinations. This finding clearly demonstrates the considerable challenge of predicting the net outcome of polyphenol mixtures on •OH formation, even under controlled in vitro conditions. The complexity increases substantially in physiological environments, such as circulating blood or intracellular compartments, where multiple factors further influence redox dynamics. These include fluctuations in pH, the presence of other redox-active metal ions (e.g., Cu^2+^/Cu^+^), endogenous low-molecular-weight antioxidants (e.g., vitamin C, glutathione), antioxidant enzymes, and the potential for phenolic compounds to form complexes with plasma or cellular proteins. Collectively, these variables render accurate prediction of either pro- or antioxidant effects of individual polyphenols, and especially their mixtures, virtually impossible in vivo. This observation warrants particular attention in light of the growing and often uncritical use of isolated polyphenolic compounds or polyphenol-rich plant extracts as dietary supplements in otherwise healthy individuals.

### 3.1. Relevance to the Development of Polyphenol Dietary Supplements

Our results could be important for the development of multi-component polyphenol supplements as antioxidants. They showed that it is almost impossible to predict resultant redox activity of a supplement composed of two or more phenolics on the basis of individual compounds activities. The situation could be more complicated when polyphenols are mixed with antioxidant vitamins [17] and some other substances to improve their absorption [18] and to enhance biosynthesis of antioxidant enzymes (e.g., selenium) [19]. Therefore, the given supplement should be tested for redox activity as a whole along with a redox characterization of its constituents at the same concentrations in the reaction mixture. However, such an approach does not completely solve this question because there are great differences between the rate of absorption of various polyphenols in the digestive tract [20], causing concentration variability of these phytochemicals in the circulating blood. In addition, endogenic production of reactive oxygen species may by modulated by polyphenols via interactions with cellular signaling proteins and enzymes involved in oxidants production [21].

### 3.2. Possible Mechanisms Responsible for Suppression of Polyphenols’ Redox Activities in Binary Mixtures

**Figure 2:**
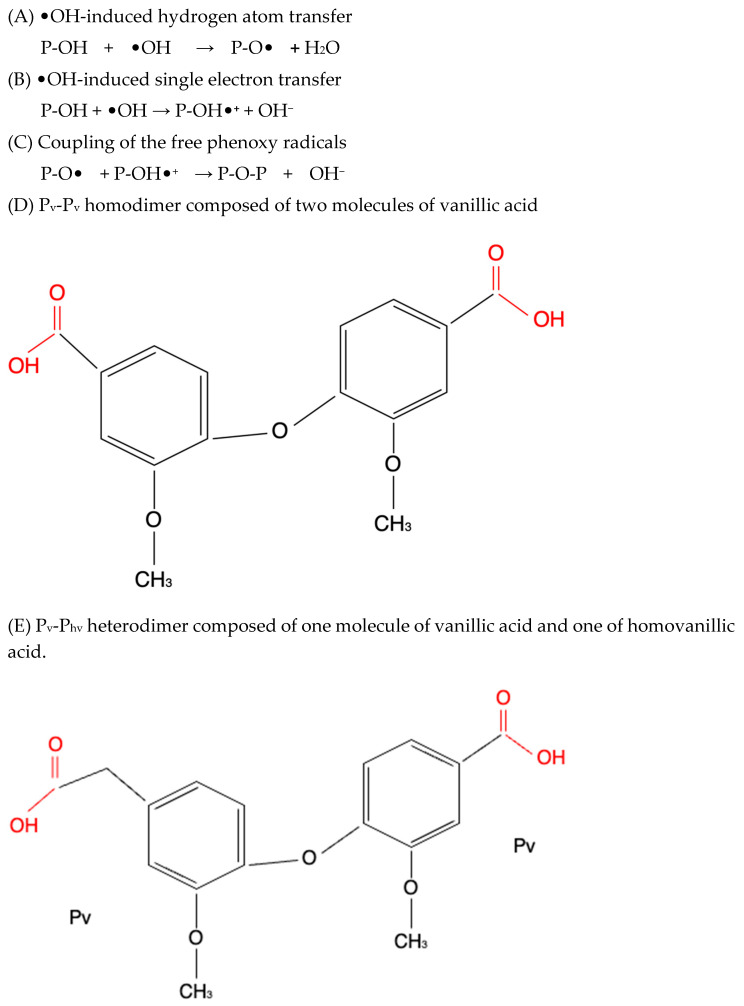
Proposed simplified mechanism of oxidative polymerization of phenolic acids induced by •OH generated within the Fe^2+^–EGTA–H_2_O_2_ system. The process involves mutual chemical interactions between phenoxy-type radicals with reduced reactivity, formed via either hydrogen atom transfer (HAT) (**A**) or single electron transfer (SET) (**B**). Subsequent radical–radical coupling results in the formation of dimeric or oligomeric products (**C**–**E**). These reactions effectively terminate the radical cascade by stabilizing the reactive intermediates through covalent bonding. P refers to a general phenolic molecule, Phv denotes homovanillic acid, and Pv corresponds to vanillic acid. In both cases, the resulting dimers possess two chelating centers (indicated in red), which may facilitate further iron-induced polymerization.

**Figure 3:**
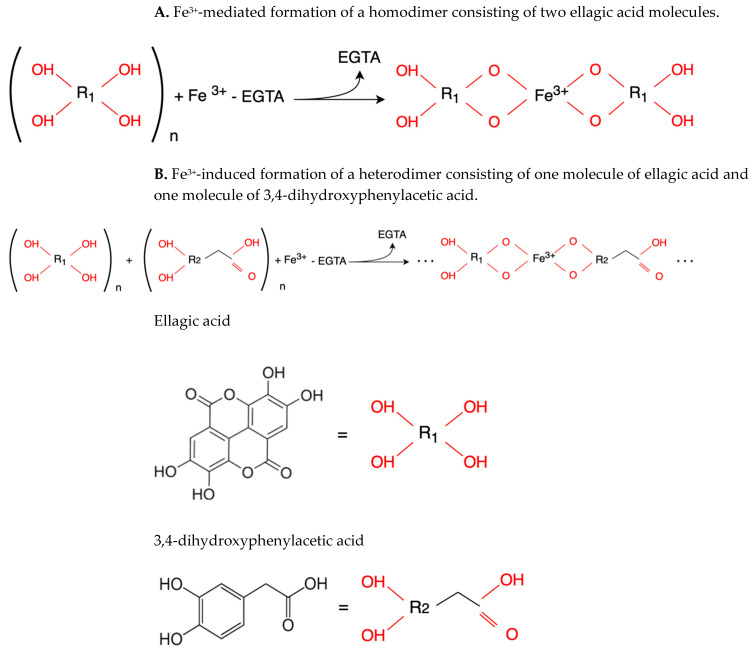
Proposed simplified mechanism of Fe^3+^-induced polymerization of phenolic acids possessing two metal-chelating centers within their backbone structure, in the context of the modified Fenton system (Fe^2+^–EGTA–H_2_O_2_). Hydrogen peroxide oxidizes Fe^2+^–EGTA to Fe^3+^–EGTA, from which a portion of Fe^3+^ ions may subsequently be displaced by polyphenolic compounds with high chelation affinity (e.g., ellagic acid or 3,4-dihydroxyphenylacetic acid).

Although we did not perform experiments to elucidate the mechanism of redox activities of simple polyphenol suppression in equimolar binary mixtures, polyphenol oligomerization (or polymerization) may be responsible for this phenomenon. This involves oxidative polymerization of phenolic acids induced by •OH generated within the Fe^2+^–EGTA–H_2_O_2_ system (Figure 2) and Fe^3+^-induced polymerization of phenolic acids possessing two metal-chelating centers within their backbone structure (Figure 3) [22,23,24]. In the first mechanism, phenoxy radicals formed after the inactivation of •OH react with each other to form dimers [22] and in the second one, phenolics having two or more chelating moieties create dimers or longer linear complexes [23,24]. The chemical structures of the five tested polyphenols are presented in Table 1. Ellagic acid, gallic acid, and 3,4-dihydroxyphenylacetic acid possess two distinct chelation sites and are thus capable of binding two iron ions simultaneously and producing oligomeric complexes (Figure 3) [23,24]. Although the polymers of polyphenols may retain functional moieties within their backbone structure that are capable of interacting with reactive oxygen species, their overall antioxidant efficiency may be significantly diminished. This is likely due to their increased molecular size and the resulting steric hindrance, which may restrict access to the critical site of the Fenton reaction between H_2_O_2_ and the Fe^2+^–EGTA complex. Strong suppression of real effect of binary mixtures containing ellagic acid in comparison to summed individual values supports this hypothesis. Moreover, the results of previous studies showing phenolic polymerization after exposition to oxidants [25] and a decrease in antioxidant activities along with increased concentration of phenolic polymers [26] are in agreement with this aforementioned hypothesis.

Methoxy group (R-O-CH_3_) is a polar functional group having a slightly positive charge on the methyl group and a slightly negative charge on the oxygen atom. It can chelate metal ions. Vanillic acid and homovanillic acid have a methoxy group; however, this group was not recognized as that involved in metal ions chelation [24]. This is due to the presence of hydroxyl group in the ortho position to methoxy group in these acids. The hydroxyl group can dissociate [27] and cause electron delocalization, thus abolishing negative charge on the oxygen atom of the methoxy group and binding of metal ions. The keto-oxygen site present in ellagic acid can bind metal cations because the oxygen atom attracts the electrons, making this site polar. On the other hand, ellagic acid has four hydroxyl groups that can dissociate (especially at pH > 7.0), being dominant structures responsible for cations complexation [24].

## 4. Study Limitations

In this study, we checked whether the predefined pro- or antioxidant activity of individual polyphenols fails or is only partially effective in predicting the net outcome of their binary mixtures on •OH-induced light emission in the Fe^2+^–EGTA–H_2_O_2_ system. The results of our experiments demonstrated significant differences between summed individual values and experimental effects of equimolar binary polyphenols mixtures on the UPE of the Fe^2+^–EGTA–H_2_O_2_ system. Moreover, based on prior observations, we proposed mechanisms that may underlie this discrepancy: polymerization of polyphenols, either as a result of cation binding [23,24] or initiated by reactions with oxidizing agents [22]. However, it is important to acknowledge that the experimental conditions described in the cited literature such as incubation time, temperature, oxidant type, phenolic concentration, and solvent composition differ substantially from those employed in the present study. As such, direct extrapolation of those findings to our system may have limitations. The most conclusive approach to validating the proposed mechanisms would involve direct detection and structural characterization of phenolic polymers formed during the Fe^2+^–EGTA–H_2_O_2_ reaction. Unfortunately, due to methodological constraints including challenges related to chromatographic column selection, mobile phase preparation, and the relatively short incubation time used, we were unable to perform such an analysis. Moreover, we only analyzed five polyphenols and six binary equimolar mixtures composed of them. This number was too small to perform a proper (with statistical confirmation, e.g., a multivariate analysis with multiple linear regressions) [28] structure–activity relationship (SAR) analysis. Another question is the relatively large variability of the results obtained. Although we used a chemiluminometer with a photomultiplier working at stable temperature 8 °C, it seems that the noise (dark current) seems to affect the UPE signal. Application of a photomultiplier working at temperature below 0 °C would probably improve the repeatability of the measurements. These represent the principal limitations of our study. Despite this, the experimental data clearly demonstrate a consistent lack of additive effects in the majority of equimolar polyphenol mixtures tested. Moreover, the observed experimental values were consistently lower than the summed individual values, strongly suggesting the occurrence of secondary chemical processes, such as iron chelation and phenolic polymerization, which result in diminished pro- or antioxidant activity of the original compounds. However, other interactions (antagonistic, additive, inhibitory) occurring simultaneously between polyphenols responsible for discrepancy among the experimental and summed individual values’ effect on the UPE of the Fe^2+^–EGTA–H_2_O_2_ system cannot be excluded.

## 5. Directions for Future Research

All issues mentioned in the previous section as limitations of the study could be recognized as justified directions of future research. However, the relevance of the Fe^2+^–EGTA–H_2_O_2_ system to physiological conditions is low and planned experiments should involve enzymatic and cellular systems responsible for oxidant production in vivo. These can include evaluation of effect of single polyphenols and their mixtures on enzymatic systems (e.g., xanthine–xanthine oxidase) [29] and resting and agonist-stimulated phagocytes, namely macrophages [30] and neutrophils [31]. On the other hand, both the antioxidant and anti-inflammatory activities of polyphenols would be tested simultaneously in this latter case.

## 6. Materials and Methods

### 6.1. Chemicals and Solutions

All chemicals used in the study were of analytical grade. Sodium phosphate monobasic monohydrate (NaH_2_PO_4_ × H_2_O), sodium phosphate dibasic heptahydrate (Na_2_HPO_4_ × 7 H_2_O), iron (II) sulfate heptahydrate (FeSO_4_ × 7H_2_O), sodium hydroxide (NaOH), and ethylene glycol-bis (β-aminoethyl ether)-N,N,N′,N′-tetraacetic acid (EGTA) were purchased from Sigma-Aldrich Chemicals (St. Louis, MO, USA). Gallic acid, ellagic acid, vanillic acid, homovanillic acid, and 3,4-dihydroxyphenylacetic acid (all of the highest available purity) were obtained from Sigma-Aldrich Chemie GmbH (Steinheim, Germany) or from Fluka, Sigma-Aldrich (Buchs, Switzerland). Hydrogen peroxide (30% *w*/*w* aqueous solution) was supplied by Chempur (Piekary Śląskie, Poland). Sterile, deionized, pyrogen-free water (freshly prepared, resistivity > 18 MΩ·cm; HPLC-grade; H_2_O Purification System, USF Elga, High Wycombe, UK) was used in all procedures throughout the study. Working solutions of 5 mmol/L FeSO_4_, 10 mmol/L EGTA, and 28 mmol/L H_2_O_2_, as well as 10 mmol/L phosphate buffer (pH 6.6), were freshly prepared immediately prior to use, according to previously described protocols [16].

### 6.2. Preparation of Aqueous Solutions of Polyphenols

According to data retrieved from the PubChem database, the maximum aqueous solubility of the polyphenols selected for this study is as follows: vanillic acid—8.9 mmol/L; homovanillic acid—93.3 mmol/L; 3,4-dihydroxyphenylacetic acid—23.7 mmol/L; gallic acid—6.5 mmol/L; and ellagic acid—3.3 mmol/L. Two sets of freshly prepared aqueous stock solutions of each polyphenol were utilized for the experiments: Set I with a final concentration of 5.8 µmol/L and Set II with a concentration of 11.6 µmol/L, both prepared in 10 mmol/L phosphate buffer at pH 6.6. Given that these working concentrations were several orders of magnitude lower than the maximum solubility limits of the respective compounds, the likelihood of precipitation in any of the stock solutions was deemed negligible.

### 6.3. Evaluation of the Effect of Single Polyphenols and Their Combination (Two Compounds) on UPE of Fe^2+^–EGTA–H_2_O_2_ System

In our previous study, the highest ultra-weak photon emission (UPE)-to-noise ratio was recorded for the Fe^2+^–EGTA–H_2_O_2_ system in 10 mmol/L phosphate buffer at pH 6.6 [16]. Accordingly, this buffer was employed in the current experiments. To ensure methodological consistency and eliminate potential sources of error, an appropriate set of controls was incorporated. Working solutions of polyphenol pairs were freshly prepared prior to use by mixing equal volumes (470 µL each) of two distinct stock solutions (11.6 µmol/L each), yielding final concentrations of 5.8 µmol/L for each compound in the reaction mixture. The experimental design, including all control conditions, is summarized in Table S2. Measurements of total light emission were conducted using a multitube luminometer (AutoLumat Plus LB 953, Berthold, Germany), equipped with a Peltier-cooled photon counter (spectral range: 380–630 nm), operating at 8 °C to ensure high sensitivity and low background noise. Briefly, 20 µL of 10 mmol/L EGTA was added to a Lumi Vial Tube (5 mL, 12 × 75 mm; Berthold Technologies, Bad Wildbad, Germany) containing 940 µL of phosphate buffer (pH 6.6). Subsequently, 20 µL of a 5 mmol/L FeSO_4_ solution was added. After gentle mixing, the tube was placed in the luminometer and incubated in the dark at 37 °C for 10 min. Then, 100 µL of a 28 mmol/L H_2_O_2_ solution was automatically injected, and total light emission (expressed in relative light units, RLU) was recorded over 120 s (serving as the complete modified Fenton system without phenolic compound). In experimental samples, either individual polyphenols or their equimolar mixtures were added (in 940 µL of phosphate buffer) to the reaction vial, followed by 20 µL of EGTA solution, 20 µL of FeSO_4_ solution, and automatic injection of 100 µL of H_2_O_2_. Additional control conditions included incomplete systems containing Fe^2+^–H_2_O_2_ or Fe^2+^–EGTA, with or without polyphenols (Table S2). All experimental conditions were tested in at least five independent replicates.

The percentage inhibition and enhancement of UPE by a given phenolic or equimolar mixture of two corresponding phenolics (experimental value) was calculated using the following formulas: percentage of inhibition = [(UPE of Fe^2+^–EGTA–H_2_O_2_) − (UPE of phenolic/phenolics − Fe^2+^–EGTA–H_2_O_2_)]/[(UPE of Fe^2+^–EGTA–H_2_O_2_) − (UPE of Fe^2+^–EGTA–H_2_O)]; percentage enhancement = [(UPE of phenolic/phenolics − Fe^2+^–EGTA–H_2_O_2_) − (UPE of Fe^2+^–EGTA–H_2_O_2_)]/[(UPE of Fe^2+^–EGTA–H_2_O) − (UPE of Fe^2+^–EGTA–H_2_O)]. In one set of experiments, all the samples (1–6; Table S2) were analyzed simultaneously to minimize the inter-assay variability.

### 6.4. Statistical Analyses

The results both theoretical (summed individual values) and experimental percentages of inhibition or enhancement of UPE (total light emission) induced by equimolar mixtures of two phenolic compounds are presented as means with standard deviations, as well as medians with interquartile ranges (IQRs). Statistical comparisons between summed individual values and corresponding experimental effects on UPE generated by the Fe^2+^–EGTA–H_2_O_2_ system were performed using either the independent-samples (unpaired) *t*-test or the Mann–Whitney U test, depending on the distribution of the data, which was assessed via the Kolmogorov–Smirnov–Lilliefors test. The equality of variances between groups was evaluated using the Brown–Forsythe test. If heterogeneity of variances was identified, Welch’s *t*-test was applied instead of the standard *t*-test. A *p*-value of less than 0.05 was considered statistically significant.

## 7. Conclusions

We found that the redox activities (summed individual values) of simple plant polyphenols are suppressed in binary mixtures of the compounds tested in •OH radicals generating modified Fenton’s system Fe^2+^–EGTA–H_2_O_2_. This shows that it is difficult or even impossible to predict net effect of a polyphenol mixture on the basis of anti- or pro-oxidant activity of individual phytochemicals. It seems completely impossible under more complex in vivo conditions like circulating blood or intracellular fluid. We propose Fe^3+^- and oxidant-induced polyphenol oligomerization as processes responsible for this phenomenon. However, confirmation of these hypotheses requires further detailed studies.

## Figures and Tables

**Table 1:** The chemical structures of the five investigated polyphenols. Each of these compounds contains at least one chelating moiety (highlighted in red), which is capable of coordinating iron ions.

Polyphenol	Chemical Structure with Marked in Red Structures Binding Iron	Name of Phenolic Chelating Group
Gallic acid	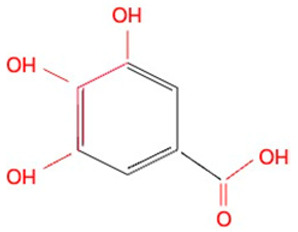	Catechol groupGalloyl group
Vanillic acid	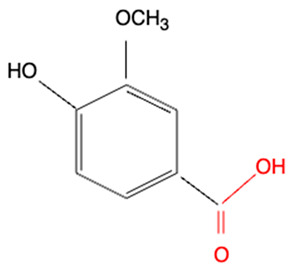	Carboxylate group
Homovanillic acid	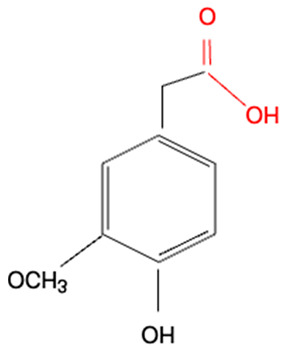	Carboxylate group
3,4-Dihydroxyphenylacetic acid	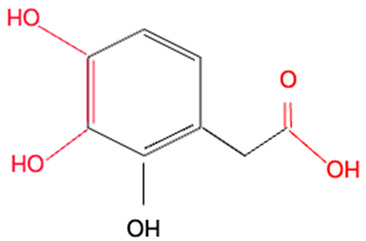	Carboxylate groupTwo hydroxyl groups
Ellagic acid	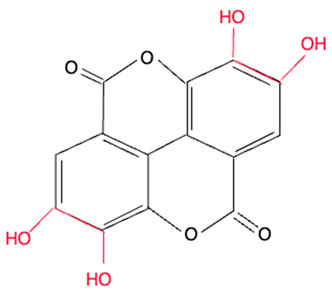	Four hydroxyl groups

## Data Availability

The data supporting the findings of this study are available upon request from the corresponding author.

## Supplementary Materials

The following supporting information can be downloaded at: https://www.mdpi.com/article/10.3390/molecules30112269/s1, Table S1: Comparison of experimental and summed individual values of binary equimolar mixtures of polyphenols on light emission (UPE) generated by the Fe^2+^–EGTA–H_2_O_2_ system; Table S2: Experimental design for the assessment of individual polyphenols and equimolar binary combinations (final concentration of each compound in the reaction milieu: 5 µmol/L) on ultra-weak photon emission (UPE) generated by the Fe^2+^–EGTA–H_2_O_2_ system.
