# Supplementary material for: A Modified Fenton’s System Fe2+–EGTA–H2O2 Reveals That Redox Activities of Simple Polyphenols Are Suppressed in Binary Mixtures
Source: Molecules. 2025 May 22;30(11):2269. doi: 10.3390/molecules30112269 (PMC12156245; doi:10.3390/molecules30112269)
Supplement: Supplementary file 1 [file molecules-30-02269-s001.zip › molecules-3608748-supplementary.pdf]

Table S1. Comparison of experimental and summed individual values of binary equimolar mixtures of polyphenols on light emission (UPE) generated by the Fe^{2+}–EGTA–H_2O_2 system.

Studied polyphenols and their equimolar mixtures at final concentration of 5 μmol/L	% inhibition (-) or % enhancement (+) of light emission from Fe^{2+}-EGTA-H_2O_2 system	
	Experimental	Summed individual values
Gallic acid	259±226 (165; 80)	-
3,4-dihydroxyphenylacetic acid	-47±22 (-57 ; 4)	
Gallic acid & 3,4-dihydroxyphenylacetic acid	512±461 (364; 324)	212±222 (157; 76) *
Gallic acid	172±113 (170; 91)	
Vanillic acid	26±10 (24; 15)	
Gallic acid & Vanillic acid	117±81 (83; 92)	197±119 (201; 101)
Gallic acid	263±36 (278; 28)	
Homovanillic acid	45±16 (42; 14)	
Gallic acid & homovanillic acid	159±106 (125; 135)	308±44 (303; 49) *
Ellagic acid	952±325 (762; 310)	
3,4-dihydroxyphenylacetic acid	-54±6 (-51; 8)	
Ellagic acid & 3,4-dihydroxyphenylacetic acid	12±15 (11; 8)	871±325 (713; 306) *
Ellagic acid	1604±286 (1547; 109)	
Homovanillic acid	85±14 (80; 22)	
Ellagic acid & Homovanillic acid	357±50 (355 ; 61)	1689±293 (1627 ; 76)*
Vanillic acid	-14±14 (-15; 18)	
Homovanillic acid	99±51 (91; 60)	
Vanillic acid & Homovanillic acid	-34± 8 (-35; 8)	85± 54 (76; 50) *

In one experiment two polyphenols and their equimolar mixture were studied. Two single polyphenols or their equimolar mixture were mixed with EGTA and Fe^{2+} and then H_2O_2 was automatically injected with subsequent measurement of total light emission. Results expressed as mean and standard deviation (median: interquartile range) of % enhancement (+) or inhibition (-) of light emission were obtained from at least 5 separate experiments. Summed individual values were calculated on the basis of assumption of summation of separate activities of tested polyphenols present in the binary mixture. * - versus corresponding experimental value – $p<0.05$.

Table S2. Experimental design for the assessment of individual polyphenols and equimolar binary combinations (final concentration of each compound in the reaction milieu: 5 μmol/L) on ultra-weak photon emission (UPE) generated by the Fe^{2+}–EGTA–H_2O_2 system.

No	Sample	Volumes of Working Solutions Added to Luminometer Tube (μL)					
		A<br>PB	B<br>Polyphenol/s*<br>in PB	C<br>EGTA	D<br>$FeSO_4$	E<br>H_2O_2	F<br>H_2O
1	Complete system	940	-	20	20	100	-
2	Complete system + polyphenol/s	-	940	20	20	100	-
3	Incomplete system	960	-	-	20	100	-
4	Incomplete system + polyphenol/s	20	940	-	20	100	-
Additional controls							
5	Fe^{2+}-EGTA without H_2O_2	940	-	20	20	-	100
6	Fe^{2+}-EGTA without H_2O_2 + Polyphenol	-	940	20	20	-	100

Working solutions were mixed in the following alphabetical sequence: A – 10 mmol/L phosphate buffer (PB, pH = 6.6); B – individual polyphenol or equimolar binary mixture of two compounds (prepared from working solutions I and II in PB, final concentration 5.8 μmol/L); C – 10 mmol/L aqueous solution of EGTA; D – 5 mmol/L aqueous solution of $FeSO_4$. After gentle mixing, each reaction tube was placed in the luminometer chamber and incubated for 10 min. at 37 °C in the dark. Subsequently, either E – 28 mmol/L H_2O_2 or F – distilled water was automatically injected using a programmable dispenser, and total light emission (UPE) was recorded over a period of 2 minutes. In each experimental series, two individual polyphenols and their equimolar combination were assessed under identical conditions.
